# Assessing the Efficacy of the Spectrum-Aided Vision Enhancer (SAVE) to Detect Acral Lentiginous Melanoma, Melanoma In Situ, Nodular Melanoma, and Superficial Spreading Melanoma: Part II

**DOI:** 10.3390/diagnostics15060714

**Published:** 2025-03-13

**Authors:** Teng-Li Lin, Riya Karmakar, Arvind Mukundan, Sakshi Chaudhari, Yu-Ping Hsiao, Shang-Chin Hsieh, Hsiang-Chen Wang

**Affiliations:** 1 Department of Dermatology, Dalin Tzu Chi General Hospital, No. 2 Min-Sheng Rd., Dalin Town, Chiayi 62247, Taiwan; tanglilin1121@hotmail.com; 2 Department of Mechanical Engineering, National Chung Cheng University, 168 University Rd., Min Hsiung, Chiayi 62102, Taiwan; karmakarriya345@gmail.com (R.K.); d09420003@ccu.edu.tw (A.M.); 3 Department of Computer Science, Sanjivani College of Engineering, Station Rd, Singapur, Kopargaon 423603, Maharashtra, India; sakshichaudharicomp@sanjivanicoe.org.in; 4 Department of Dermatology, Chung Shan Medical University Hospital, No. 110, Sec. 1, Jianguo N. Rd., South Dist., Taichung City 40201, Taiwan; missyuping@gmail.com; 5 Institute of Medicine, School of Medicine, Chung Shan Medical University, No. 110, Sec. 1, Jianguo N. Rd., South Dist., Taichung City 40201, Taiwan; 6 Division of General Surgery, Department of Surgery, Kaohsiung Armed Forces General Hospital, 2 Zhongzheng 1st. Rd., Lingya District, Kaohsiung City 80284, Taiwan; 7 Hitspectra Intelligent Technology Co., Ltd., Kaohsiung 80661, Taiwan

**Keywords:** skin cancer, acral lentiginous melanoma, melanoma in situ, nodular melanoma, superficial spreading melanoma, hyperspectral imaging, band selection, spectrum-aided visual enhancer

## Abstract

**Background:** Melanoma, a highly aggressive form of skin cancer, necessitates early detection to significantly improve survival rates. Traditional diagnostic techniques, such as white-light imaging (WLI), are effective but often struggle to differentiate between melanoma subtypes in their early stages. **Methods:** The emergence of the Spectrum-Aided Vison Enhancer (SAVE) offers a promising alternative by utilizing specific wavelength bands to enhance visual contrast in melanoma lesions. This technique facilitates greater differentiation between malignant and benign tissues, particularly in challenging cases. In this study, the efficacy of the SAVE is evaluated in detecting melanoma subtypes including acral lentiginous melanoma (ALM), melanoma in situ (MIS), nodular melanoma (NM), and superficial spreading melanoma (SSM) compared to WLI. **Results:** The findings demonstrated that the SAVE consistently outperforms WLI across various key metrics, including precision, recall, F1-scorw, and mAP, making it a more reliable tool for early melanoma detection using the four different machine learning methods YOLOv10, Faster RCNN, Scaled YOLOv4, and YOLOv7. **Conclusions:** The ability of the SAVE to capture subtle spectral differences offers clinicians a new avenue for improving diagnostic accuracy and patient outcomes.

## 1. Introduction

Skin cancer is said to be one of the most commonly occurring cancers with a rapidly increasing incidence. It is mainly classified into two main types, malignant melanoma (NM) and non-melanoma skin cancer (NMSC) [1]. However, the rate of incidence of malignant melanoma is far higher than NMSC melanoma [2], and thus, the mortality rate is higher in melanoma [3]. Research has shown that melanoma is responsible for around 55,500 deaths annually, which represents up to 0.7% of the mortality occurring due to all cancers collectively [4]. The major threat factor for melanoma is UV radiation from sun exposure [5]. The penetration of UV radiation is much higher in light-skinned people (SPF 3.3) than in people with colored skin (SPF 13.4) [6], threatening the former with skin cancer. In 2020, around 324,635 new melanoma cases were estimated, while 57,043 mortalities were accounted for. According to the World Health Organization (WHO), around 132,000 patients are diagnosed with melanoma globally every year [7]. Furthermore, it can be divided into four main subtypes: superficial spreading melanoma (SSM), nodular melanoma (NM), lentigo malignant melanoma (LMM), and acral lentiginous melanoma (ALM).

These subtypes are also gender-biased as SSM and LMM prevail more in females, while ALM and NM are said to occur more commonly in males [8]. SSM is said to be the most common subtype of malignant melanoma [9] with around 70% of cases falling under it. Previous research claims that round junctional nevus cells give rise to SSM, while spindle-shaped junctional melanocytes are responsible for LLM [10,11]. LLM mainly targets the head area and neck area of the body [12], along with the possibility of cheeks. A noteworthy behavior of LLM is its tendency to regression [13]. Patients who were diagnosed with LLM previously noticed that the hyperpigmentation caused by LLM was reverting to either white color or their original skin color. NM is said to be the most aggressive type because of the number of deaths it causes. In December 2022, a unique case of NM growing over SSM was discovered on the trunk of a 59-year-old man from Syria [14]. ALM is the rarest subtype of melanoma [15]. It occurs more on the body parts that are thick-skinned like palms, soles, and nails. Jung et al. (2013) described that the dominant places of ALM are the thumbnails and first finger/toe nails [16].

Melanoma needs to be detected early as it is the most aggressive type of skin cancer. The conventional method for the detection of skin cancer is biopsy (either punch or shave) [17]. This method is often painful time, consuming, and requires more resources. Therefore, the use of artificial intelligence and machine learning (AI ML) is prominent. Vidya et. al. classified skin lesions between melanoma and benign using the K-neared neighbor (KNN) and Naïve Bayes classifier algorithms and obtained an accuracy of around 97.8% on 672 melanoma and 328 benign images [18]. In contrast, Monika et al. used a multiclass support vector machine (MSVM) on 25,000 images of eight different types of dermoscopic images and obtained an accuracy of 96.25% with a precision of 96.32% [19]. In another paper, Das et al. obtained an accuracy, specificity, and sensitivity of 98%, 94%, and 92%, respectively, with a deep convolutional neural network (CNN) model on 595 lesions to detect melanoma from nevi [20]. Although the RGB method for the detection of skin cancer is better, drawbacks like different lighting conditions and less color contrast may mislead observers in terms of noticing subtle color differences. Spectral information goes beyond the information provided by RGB images and is a crucial part of analyzing any substance. Hyperspectral imaging (HSI) provides us with this continuous spectral information with each image, which is not possible with mere RGB images [21].

HSI is an advancing technique that captures images across a wide spectrum wavelength that creates 2D spectral images and a 3D data cube, further, representing spectral and spatial information. HSI provides much more information than RGB and it can be seen as a high-dimensional vector in the dimension of the spectrum [22]. HSI acquires its data from a 3D data cube which contains the information in two dimensions for spatial information and one dimension for spectral information [23], furthermore, the analysis and processing of the data cube is performed to extract valuable and needed data from it. Gamal ElMasry et al. listed some advantages of HSI over traditional techniques that include faster data recording, and nominal preparation of samples [24]. Crop monitoring [25], soil analysis [26], water quality prediction [27], air pollution detection [28], and target detection [29] are some of the applications of HSI. The working of HSI is based on mainly 4 principles: spectral scanning (push broom scanning), spatial scanning (whiskbroom scanning), snapshot imaging, and medical HSI system [30]. Detection of cancer at an early stage is nearly impossible with bare RGB imaging [31]. Therefore, HSI is combined with narrow-band imaging (NBI) that enhances the images, leading to better results.

NBI was first developed by Olympus System, Tokyo and it was initially developed majorly to enhance the visibility of mucosal microvascular structure [32]. Commonly used for endoscopy, NBI filters out the WLI into colors of shorter wavelength, majorly: blue spectrum (415 nm) and green spectrum (540 nm) [33], as blue light is absorbed more by red blood cells and green light goes deeper into the tissue. Hemoglobin shows a maximum absorption of 415 nm and hence, blue light is absorbed strongly by it [34]. These lights penetrate less as compared to red light (750 nm) causing a black/brown colored contrast in the top layer of tissues, hence facilitating analyses of the superficial microvessels more efficiently [35]. Unlike RGB imaging which uses a broad spectrum of light, NBI uses modified narrow bands of light [36] that make it easier to differentiate the most subtle differences in mucosal tissues and blood vessels, in our case, it shows the most subtle difference in the vascular patterns of skin.

The motivation for this study lies in the need for early and accurate diagnosis of subtypes of melanoma. RGB lacks the spectral depth that is required for accurate diagnosis of melanoma, leading to inaccurate detection. This resulted in prompting the exploration of the Spectrum-Aided Vision Enhancer (SAVE). The SAVE consists of broader spectral information. The SAVE’s imaging techniques are combined with ML algorithms in this study, overcoming the limitations put by RGB. The results of the study not only showcase how the SAVE outperforms WLI but also open the scope of discussion for the SAVE’s application in other medical conditions, where high-resolution imaging plays a crucial role in diagnosis. This study uniquely combines the SAVE with several advanced deep learning models (YOLOv10, Scaled YOLOv4, YOLOv7, and Faster R-CNN) to enhance melanoma subtype detection, a topic that has not been thoroughly investigated in previous research. In contrast to conventional RGB imaging and other spectrum techniques, the SAVE markedly improves the distinction of melanoma, which exhibit reduced detection accuracy with current approaches. This study thoroughly compares the SAVE with WLI across many performance criteria (precision, recall, F1 score, and mAP), providing a comprehensive assessment of its therapeutic relevance. Our study presents compelling evidence for the prospective implementation of the SAVE in real-world clinical settings by illustrating its capability to diminish false positives while preserving high sensitivity.

## 2. Materials and Methods

### 2.1. Dataset

**Figure 1:**
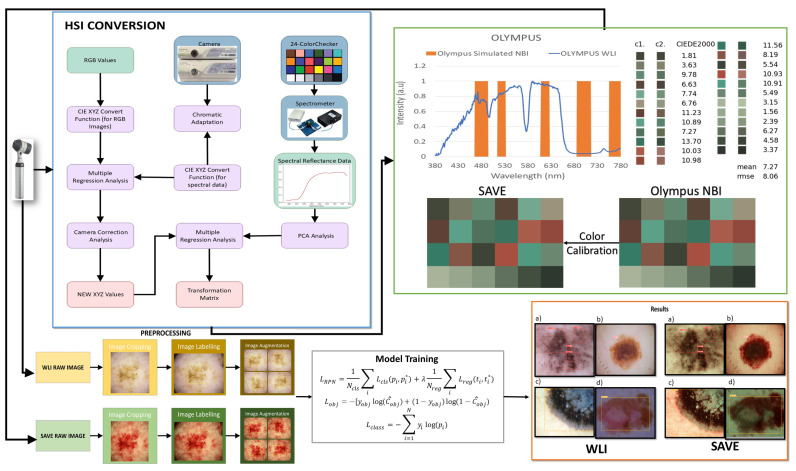
Flowchart of the whole study.

The dataset for melanoma subtypes were provided by Dalin Tzu Chi Hospital, Minsheng Rd, Dalin Township, Chiayi County, Taiwan. The dataset originally consisted of 882 images, randomly distributed amongst ‘ALM’, ‘SSM’, ‘M in Situ’, and ‘NM’. ALM consists of 343 images, SSM 254 with images, M in Situ with 184 images, and NM with 101 images. This work employed a high-quality, clinically validated dataset, thereby augmenting the reliability of melanoma diagnosis relative to open-source datasets. This hospital-specific dataset guarantees standardized imaging circumstances and precise ground truth annotations, in contrast to open-source available datasets that may exhibit variations in image capture and labeling. This enhances the robustness of the SAVE evaluation, rendering the findings more clinically pertinent and applicable to real-world melanoma detection. A software package named ‘LabelImg’ v 1.8.6 was used for annotating each image with its class name. The dataset was annotated in two formats for this study: COCO format for YOLOv10 and Faster R-CNN, and Scaled-yolo format for Scaled YOLOv4 model. The dataset was pre-processed to the fixed resolution of 640 × 640 pixels so as to avoid inconsistencies with YOLO architecture. It was further augmented with augmentation techniques like 90° clockwise and anticlockwise rotation, 15° shear rotation, and horizontal and vertical flips. Color-based augmentations are strictly avoided because the study involves the comparison based on the performances of WLI and the SAVE. The dataset was randomly split into the train, valid, and test image sets in the ratio of 7:2:1, respectively. The models were set to run for 600 epochs with a batch size of 16. The Intersection over Union (IoU) is set as 0.5 for training and 0.65 for validation. Confidence is set to 0.001, while the learning rate is 0.01. A previous study [37] shows how the ‘Stochastic Gradient Descent’ (SGD) algorithm is better at generalization than Adam, and therefore, SGD is used as an optimizer. All the images from each class are distributed in either close-up images, dermoscopy images, or clinical images. Although there are some concerns with regard to the difference in the number of images per modality, the model is trained in this manner so that no one modality influences the results more than others. This implies employing augmentation and normalization procedures so that the invasiveness of native features is restricted. The architecture of the model has a wide variety of images from different modalities which makes it easier to perform broader generalization across many sources of images. The diversity of the images proves rather effective as it equips model to detect real-world instances of skin cancer. The evaluation metrics such as precision, recall, F1 score, and mAP are calculated through detailed validation and testing across all modalities. The overall schematics of the research is shown in Figure 1.

### 2.2. SAVE

Any color that the human eye perceives can be represented using RGB values. The various combinations of RGB correspond to a different color. In the case of HSI, the colors are based on these values along with the intensity of light absorbed and reflected. The SAVE method comprises the conversion of colors present in the RGB image taken by a digital camera to an HSI image by deriving its reflectance chart. Therefore, the Macbeth Color Checker, also known as X-Rite Classic, contributes to the process of calibration. X-Rite Classic is a popular tool consisting of 24-color patches, including primary colors (red, green, and blue), secondary colors (cyan, magenta, and yellow), and six shades of gray. The images in the 24-color patch are converted to CIE 1931 XYZ color space that normalizes the RGB values to a lesser gamut and further linearizes them to CIE 1931 color space, to correctly perceive the colors based on human vision. The images captured by the digital camera may be affected by some error or noises; therefore, to correct the error, we use a variable matrix as shown in Equation (1):

(1) $$[C]=[{XYZ}_{Spectrum}]\;\times \;\mathrm{pinv}([V])$$

After correction, the new *X*, *Y*, and *Z* values were calculated by Equation (2):

(2) $$\left[{XYZ}_{Corrcnt}]=[C]\times [V\right]$$

The algorithm translates the colors from the camera and the spectrometer into the *XYZ* color space. To convert sRGB to the *XYZ* color gamut space on the camera part, the spectrometer’s convergence of reflectance spectra into the *XYZ* color space is given by Equations (3), (4), (5), and (6), respectively:

(3) $$X=k\int_{400\;\mathrm{nm}}^{700\;\mathrm{nm}}S(\lambda )R(\lambda )\bar{x}(\lambda )d\lambda$$

(4) $$Y=k\int_{400\;\mathrm{nm}}^{700\;\mathrm{nm}}S(\lambda )R(\lambda )\bar{y}(\lambda )d\lambda$$

(5) $$Z=k\int_{400\;\mathrm{nm}}^{700\;\mathrm{nm}}S(\lambda )R(\lambda )\bar{z}(\lambda )d\lambda$$

(6) $$k=100/\int_{400\;\mathrm{nm}}^{700\;\mathrm{nm}}S(\lambda )R\bar{y}(\lambda )d\lambda$$

A fixed value represents the dark current component of the imaging device. By standardizing the *V*_color_ and *V*_Non-linear_ product along with *V*_Dark_, we obtain the variable matrix *V*, and this standardization is limited up to the third order to avoid the case of over-correction. A device named Ocean Optics QE65000 is used along with a 24-color patch reflectance spectrum (X-rite board) for color transformation into the *XYZ* color space. Initially, the spectrometer measures the colors on a 24-color patch board to achieve *XYZ* values. Regression analysis process was used to establish a precise mathematical relationship, minimizing errors in color space conversion which helped optimize the transformation matrix (*M*) by adjusting for sensor-specific deviations. A second regression analysis was applied to adjust for sensor-specific deviations, improving the alignment of estimated *XYZ* values with reference spectrometer data.

(7) $$\left[M]=[Score]\times pinv([V_{Color}]\right)$$

A transformation matrix for colors is then developed with the help of reflectance spectrum data (R_spectrum_). Score represents the similarity measure used to evaluate the accuracy of the transformation between the estimated *XYZ* values (from camera RGB data) and the reference *XYZ* values (from spectrometer data). Further, a principal component analysis (PCA) is applied to R_Spectrum_ to recognize 6 principal components, also known as PCs, that successfully justified 99.64% of the information. The transformation matrix from sRGB to *XYZ* utilized in this study was predominantly based on typical CIE 1931 transformation values. To verify the accuracy, in particular in our imaging system, an experimental calibration was performed with a color checker under regulated lighting circumstances. This calibration refined the transformation matrix by considering camera sensor features and spectral response variances, ensuring consistency in color space conversion for the enhanced analysis of the SAVE images. A transformation matrix was created and was correlated with the PCA components, which resulted in a very low RMSE value of 0.056 and color difference of 0.75 indicating high similarity in colors. This method efficiently converts RGB images captured into HSI images. After calibration, the average chromatic aberration lowers from 10.76 to 0.63, giving a significant improvement in color accuracy. The reflectance differences between major colors like red, green, blue, yellow, cyan, and magenta were derived, and the results showed that red exhibited the highest deviation in longer wavelengths between 600 and 780 nm, indicating this as a limitation of this study. The other 23 color blocks had an RMSE value of less than 0.1, black being the one with the smallest RMSE value of 0.015 and the average RMSE being 0.056, proving high color reproduction accuracy. When the RMSE values were visually and numerically represented, the mean color difference was found to be 0.75, indicating the visual accuracy of color reproduction.

The detection of skin cancer is difficult with WLI; hence, bands of particular wavelengths are used to enhance the affected area, making it easier to detect skin cancer in its earlier stages. This technique utilizes HSI conversion to convert RGB images to NBI that can be used in Olympus cameras to detect esophageal cancer. It ensures that there is a negligible difference between the images generated by this algorithm and the NBI images captured by the Olympus endoscope. For this, color calibration is performed which uses the same 24-color checker. The CIEDE 2000 color difference was evaluated and resulted in a negligible value of 2.79. After the color between the SAVE-generated and real NBI is matched, primarily three major factors that contribute to color difference are taken into consideration: the color matching function, the light function, and the reflection spectrum. A significant difference in the intensity of wavelengths in the 450–540 nm range was noticed, as this is where most of the light is absorbed. This light spectrum was calibrated using Cauchy–Lorentz visiting distribution, along with the annealing optimization function given by Equation (8):

(8) $$f\left(x;x_{0},\gamma \right)=\frac{1}{\pi \gamma \;[1+{\left(\frac{x-x_{0}}{\gamma }\right)}^{2}]}=\frac{1}{\pi }\;[\frac{\gamma }{{\left(x-x_{0}\right)}^{2}+\gamma^{2}}]$$

This function simplifies classical simulated annealing (CSA) into fast annealing (FSA). The color difference was hence reduced to a negligible value of 5.36. Although the peak hemoglobin absorption level was noted at 415 nm and 540 nm, traces of brown shade corresponding to the wavelength of 650 nm were also captured in the real NBI image by the Olympus endoscope. Thus, additional wavelengths including 600 nm, 700 nm, and 780 nm were also included in the calibration process in order to enhance skin cancer detection, which accounted for small post-processing effects. This enhances the resemblance of actual NBI images with the calibrated images. The structural similarity index (SSIM) for the SAVE images increased to 94.27%, while entropy averaged 0.37%. The peak signal-to-noise ratio (PSNR) of the Olympus images was 27.88 dB, validating the accuracy of the spectral conversion algorithm and its application in medical imaging.

### 2.3. Machine Learning Architectures

This study selected Faster R-CNN, YOLOv10, Scaled YOLOv4, and YOLOv7 for melanoma detection because of their equilibrium between precision and velocity. Faster R-CNN guarantees superior detection accuracy, but YOLO-based models offer real-time processing capabilities, crucial for clinical applications. These models have demonstrated exceptional efficacy in medical imaging, especially in dermatology, rendering them suitable for assessing the SAVE. Their selection guarantees a thorough and dependable comparison analysis. Each deep learning model was trained independently on the WLI and SAVE datasets to ensure an unbiased and fair comparison of their performance. No mixed training was conducted, and the evaluation was performed separately for each imaging modality. This approach facilitates a direct assessment of the impact of the SAVE compared to RGB imaging in melanoma detection.

#### 2.3.1. YOLOv10

YOLOv10 was introduced by Wang et al. [38] in May 2024 (Tsinghua University) and delivers good accuracy with optimal computational efficiency. A novel approach known as Consistent Dual Assignments was introduced in YOLOv10 for the non-maximum suppression (NMS) free approach, which is responsible for increasing precision in object detection by reducing the number of false positives. YOLOv10 proves to be superior because of its capabilities like improved object detection for smaller objects and a reduction in number of false positives. YOLOv10 offers flexibility in choosing the model sizes (n, s, m, l, x) according to the required application. The network structures of YOLOv8 and YOLOv10 are similar [39].

The YOLO structure separates the classification and detection head. The classification head assigns a Binary Cross-Entropy loss given by Equation (9):

(9) $${Loss}_{BCE}=-w[y_{n}\log\log x_{n}+(1-y_{n})\log\log(1-x_{n})]$$

where ‘*w*’ is the weights, ‘*y*’ is the label, and ‘*x*’ is the predicted value generated by the model. The regression branch on the other hand combines Distributional Focal Loss (DFL) and CIoU loss, given by Equation (10):

(10) $${Loss}_{DF}=-[(y_{n+1}-y)\log\log\frac{y_{i+1}-y}{y(i+1)-y_{i}}+(y-y_{n})\log\log\frac{y-y_{i}}{y_{i+i}-y_{i}}]$$

This is achieved due to extraordinary innovations like efficiency-driven modules, continuous dual assignments for NMS-free training, and techniques that increase accuracy like Scaled Weight Shortcuts and Scaled Residual Connections [40].

#### 2.3.2. Faster RCNN

Introduced by Shaoqing Ren in 2015 [41], Faster R-CNN (Region-based Convolution Network) is one of the most used two-stage object detection models. Just like its predecessors, Faster R-CNN also uses the deep convolutional neural network to extract feature maps from input images. The dataset is given to the model’s backbone network for feature extraction, and a feature map with two paths is output using a convolution network. The first path is to enter the region proposal network (RPN) to extract the region of interest (ROI), and the second path is to map the extracted ROI to a shared feature map. This ROI is further input into classification and regression [42]. Faster R-CNN’s ability to make use of deep learning techniques to extract high-quality features makes it one of the most accurate object detection models.

Total RPN loss combines both classification and regression losses and is given by Equation (11):

(11) $$L_{RPN}=\frac{1}{N_{cls}}\sum_{i}L_{cls}(p_{i},p_{i}^{*})+\lambda \frac{1}{N_{reg}}\sum_{i}L_{reg}(t_{i},t_{i}^{*})$$

where $p_{i}^{*}$ is the ground truth label, $N_{cls}$ is the number of anchors, $L_{cls}$ is the classification loss, and $L_{reg}$ is the regression loss.

The total loss of FasterRCNN is given by combining classification and regression losses:

(12) $$L=L_{RPN}+L_{cls}+L_{reg}$$

#### 2.3.3. Scaled YOLOv4

YOLOv4 is a high-precision model that works in real time and is based on a one-stage detection module [43]. It was found that YOLOv4 tends to miss the detections of small objects by either completely missing them or incorrectly detecting them, which hinders the overall performance of the model [44]. The need for scaled YOLOv4 is raised from this very drawback. Scaled YOLOv4 is capable of achieving a good combination of accuracy and speed, and even performs well in real time [45]. Scaled YOLOv4 introduces the method of CSPDarkNet53 and PANet into its architecture, which results in better performance [46].

Classification loss of scaled YOLOv4 is given by Equation (13):

(13) $$L_{cls}=-\sum_{i}\left[y_{i}\log\hat{{(p}_{i})}+(1-y_{i})\log\left(1-\hat{p_{i}}\right)\right]$$

Confidence loss is given by Equation (14):

(14) $$L_{obj}=-[y_{obj}\log({\hat{C}}_{obj})+(1-y_{obj})\log\left(1-{\hat{C}}_{obj}\right)$$

#### 2.3.4. YOLOv7

YOLOv7 is a cutting-edge model for real-time object detection that improves upon the previous versions of YOLO concerning their strengths and weaknesses. It enhances speed and accuracy to a new level with architectural improvements using techniques such as reparameterization and layer aggregation, which facilitates faster inference with more precision. Furthermore, YOLOv7 also solves the small-sized object issue that was prevalent in the earlier models such as YOLOv4. It is ideal for many real-time detection applications due to the delicate balance between the speed and accuracy of the model, and it also surpasses the previous versions of the YOLO model in the quality of detection and speed of operations.

Classification loss of YOLOv7 is given by the following:

(15) $$L_{class}=-\sum_{i=1}^{N}y_{i}\log{(p}_{i})$$

## 3. Results

The results were derived based on a dataset that was pre-processed with image auto-orientation, resizing standard dimensions to 640 × 640 pixels. The dataset was also augmented using several augmentation techniques that led to better results. The results of the proposed three models are analyzed, by melanoma subtypes, and their performance is assessed in terms of evaluation metrics like precision (P), recall (R), F1 score, and mean average precision (mAP). The results are shown in Table 1.

**Figure 2:**
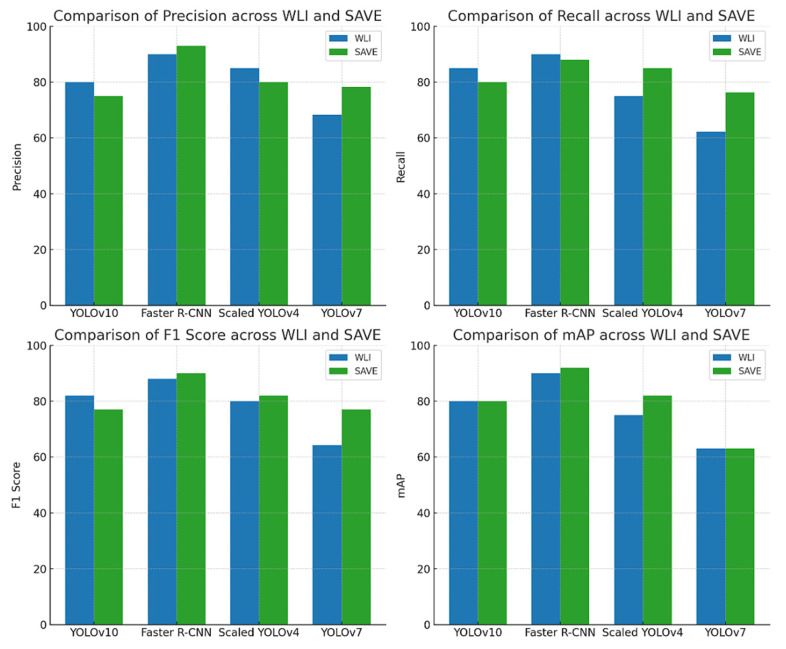
Evaluation of precision, recall, F1 score, and mAP for melanoma subtype detection across various object detection algorithms (YoloV10, Faster R-CNN, Scaled YOLOv4, and YOLOv7) using two imaging modalities: WLI and SAVE.

**Figure 3:**
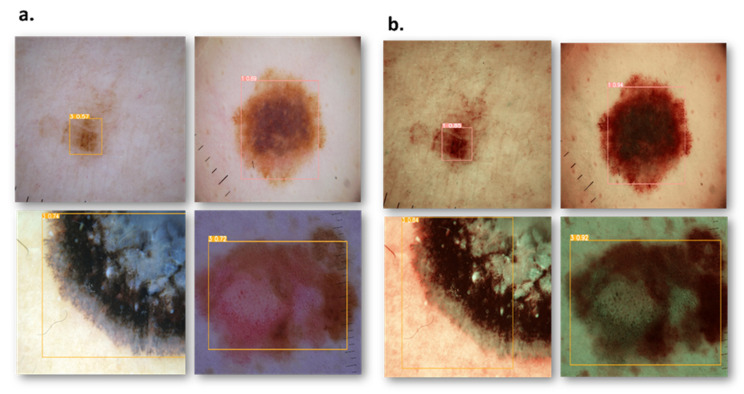
Comparison results of RGB and SAVE images show an increase in the confidence threshold in all four classes. (**a**) represents the RGB images while (**b**) represents the corresponding SAVE images.

Figure 2 shows the graphical representation of the results. In the very first model, YOLOv10, ALM did not show a significant difference between the modalities, with precision decreasing from 85% to 83%, recall increasing from 82% to 83%, and F1 score showing no change in both the modalities, 83%, proving that although WLI has marginally high precision, the SAVE’s consistency makes it equally effective (Figure S1 shows the Confusion matrix of YOLOv10: WLI and Figure S3 Confusion matrix of YOLOv10s: SAVE). The SAVE significantly outperforms WLI in the case of M in Situ with a notable increase in precision from 70% to 89% and recall from 67% to 84%, showing the SAVE to be much more reliable than WLI (Figure S2 shows the Loss graphs for YOLOv10: WLI and Figure S4 shows the Loss Graphs for YOLOv10: SAVE). The results of WLI suggest that WLI seems to be struggling to distinguish M in Situ, likely due to subtle features of M in Situ, which was enhanced in the SAVE and hence helped in achieving a better detection. For NM, again the SAVE outperforms WLI with an 8% higher precision and 17% increase in recall compared to WLI. WLI’s lower recall (69%) suggests it missed a notable number of instances of NM cases, most probably due to the different presentations of NM, which the spectral enhancement of the SAVE could detect easily. Lastly, SSM, being no exception, clearly shows the advantage of the SAVE over WLI, with the balanced performance of SAVE in both precision and recall (84% and 85%, respectively) contrasting with WLI’s lower recall (63%). mAP showed a huge increase from 81% to 93%, proving the accuracy of the SAVE to be superior. For Faster R-CNN, the results are seen as quite contradictory. ALM shows a high performance in both modalities, with the SAVE having a slightly lower recall (95% vs. 92%). The precision showed by SAVE is 100% indicating it identified no false positives (Figure S7 shows the Confusion matrix for Faster-RCNN: WLI and Figure S8: Confusion matrix for Faster R-CNN: SAVE). WLI’s slightly high recall indicates that it may be more reliable in capturing a broader range of ALM cases. In the case of M in Situ, WLI significantly outperforms the SAVE across all metrics, while WLI shows a precision of 98% against the SAVE’s precision of 90% and recall of 93% against 89%. The SAVE’s lower precision and recall suggest that it struggles more with false positives and false negatives. The difference in the F1 score shows that WLI is much more reliable than the SAVE for this specific subtype. In the case of NM, although the SAVE has a lower precision of 95% against WLI’s perfect precision of 100%, the higher recall (86%) of the SAVE suggests a more balanced performance. Despite WLI’s absolute precision, an F1 score of 88% vs. 90% suggests that the SAVE may be more effective overall in detecting NM. For SSM, WLI exhibits a strong performance in both precision and recall (94%, 95%). The SAVE, while matching in precision, has a considerably lower recall (95% vs. 77%), suggesting that WLI is more effective for SSM detection. On the other hand, for scaled YOLOv10, as far as ALM is concerned, the SAVE outperforms WLI. Although the precision is lower in the SAVE (82% vs. 80%), a notable improvement in the recall can be seen (74% vs. 84%), indicating that the SAVE is more effective than WLI for detecting ALM. Moreover, the SAVE achieves a higher mAP (78% vs. 68%) proving its consistency throughout different thresholds. For M in Situ, the SAVE outperforms WLI, by having a higher precision and recall (88% vs. 70% and 86% vs. 79%, respectively). The overall performance of mAP confirms the SAVE’s advantage for this subtype. In the case of NM, WLI achieves a slightly higher precision of 88%, but the SAVE yields a more stable F1 score by having a balanced precision and recall (82% and 80%, respectively). Finally, SSM, proving no exception, shows the SAVE’s slightly higher performance against WLI with an F1 score of 84% vs. 83%, respectively. The higher recall in the SAVE indicates it captures few more true positives than WLI, while mAP reflects the consistency in SSM detection. However, for YOLOv7, the SAVE outperforms WLI when it comes to ALM. Although the precision is slightly lower in the SAVE (73% vs. 74%), there is a notable improvement in recall (77% vs. 69%), leading to a higher F1 score (75 vs. 71), which highlights the SAVE’s effectiveness in detecting ALM. In terms of M in Situ, both precision and recall are higher for SAVE (82% vs. 67% and 78% vs. 64%, respectively), resulting in a superior F1 score of 80 compared to 65 for WLI. For NM, WLI shows a marginally better precision (70% vs. 81%); however, the SAVE maintains a more balanced performance with a higher F1 score (76 vs. 59) due to its better recall (73% vs. 52%). Lastly, for SSM, both methods perform similarly in precision and recall, but the SAVE shows a slight edge with an F1 score of 77 compared to 62 for WLI, showcasing its reliability in detecting this subtype. Considering the performance levels of all the models collectively, except for Faster R-CNN, the SAVE consistently outperforms WLI, making it a more reliable modality for melanoma detection. The SAVE’s stronger precision in challenging subtypes proves that it not only detects more instances but also does so with higher consistency. Even when the WLI exhibits higher precision, the SAVE’s balance between precision and recall ensures a more effective overall performance, proving its superiority against WLI, as shown in Figure 3 (Figure S5 shows the Results of YOLOv10: WLI and Figure S6 shows the results on YOLOv10: SAVE).

## 4. Discussion

Faster R-CNN has superior efficacy in identifying intricate lesion patterns. It employs a region proposal network (RPN) that enables concentration on small and complex melanoma characteristics, rendering it especially useful for MIS and NM, where lesion margins may be indistinct. YOLO models emphasize real-time detection. Although YOLOv10, Scaled YOLOv4, and YOLOv7 are geared for speed, they employ a single-stage detection methodology, which may occasionally undermine detection accuracy for irregularly shaped or subtle melanoma subtypes (Figure S9: Loss Graph for Scaled YOLOv4: WLI and Figure S11 Loss Graph of Scaled YOLOv4: SAVE) (Figure S10: Results of Scaled YOLOv4: WLI and Figure S12: Results of Scaled YOLOv4: SAVE). The two-stage processing of Faster R-CNN enhances its robustness in managing intricate textural characteristics, while YOLO models may exhibit superior generalization for bigger, well-defined lesions but encounter difficulties with ambiguous situations. While this study has laid a strong foundation for comparing the effectiveness of SAVE imaging over WLI in melanoma detection, several exciting future directions are worth exploring. One of which includes the preprocessing and normalization of the dataset to a fixed resolution of 640 × 640 pixels. Normalizing the dataset to a fixed resolution might be better in terms of a computational point of view and might require fewer computational resources, and it facilitates an exploration of the opportunities of adaptive resolution techniques that preserve the information of images. Further studies may explore these methods to fulfill the image quality resolution that also satisfies computational efficiency. The dataset collected for this study is from a single hospital. This issue limits the generalizability of the results and may result in a biased performance. A key factor of the optimal model performance is its diverse dataset, which is also generalizable. While this study has provided valuable insights into the dataset that is limited to a single hospital, collecting a dataset from more than one hospital may help in the robustness of the model by increasing its generalizability. Additionally, the computational resources required for the ML algorithms provide an opportunity for optimization. The complexity of the algorithm may result in high time and resource consumption. However, the ultimate goal is to develop a system that performs well with good accuracy and is well within reach with advancements in computing power. The objective of the study was to obtain accurate results in real time, and this necessitates the enhancement of the resources required. Other ways of improving real-time diagnostics will involve the development of effective data pipeline efficiency. This will be realized through data compression, smart filtering, and the prioritization of high-value data for support in the seamless handling of big datasets, with enhanced feasibility of the models in clinical settings. By understanding these tactics, this technology could be a game-changer for high-stakes diagnostics in real time, not just for melanoma, but for a wide range of conditions wherein early detection by imaging can make a real difference, including colorectal, lung, and esophageal cancers. Therefore, this study lays a strong foundation for further innovations in this area, representing a way in which future studies are supposed to be conducted to enhance methodologies and enlarge the circle of applicability. The foundation laid here ensures that future research will push the frontiers of medical imaging and machine learning even further, toward improved outcomes for patients and enhanced clinical efficiency on a global scale. Although the SAVE improves melanoma diagnosis in comparison to RGB, it possesses specific limitations when juxtaposed with conventional HSI techniques. The SAVE acquires certain wavelength bands, while HSI offers more comprehensive spectral information across an extensive range. Future research could investigate broadening the SAVE’s spectral range, incorporating deep learning-based spectral reconstruction, and creating hybrid methodologies that merge the SAVE with HSI-derived features to improve detection accuracy and spectral resolution while preserving real-time efficiency.

## 5. Conclusions

To sum up, the SAVE is a more advanced and accurate imaging technique than WLI for the diagnosis of melanoma. The SAVE has outperformed various models of YOLO, that is, YOLOv10, scaled YOLOv10, and YOLOv7, by offering the optimal ratio of precision to recall. This ratio is important in increasing the true positive rate while reducing the incidence of false positives; therefore, the SAVE is suitable for clinical circumstances in which both metrics have to be precise and accurate. The SAVE is also very reliable in detecting advanced melanoma subtypes such as M in Situ and especially NM as opposed to WLI, which exhibits low detection rates in those areas. The added spectral features that come with the SAVE allow it to distinguish elements that WLI is not able to, thus greatly enhancing both the recall and precision. This is exemplified by the higher-than-average primary metrics such as F1 score and mAP, whereby SAVE is capable of spotting melanomas with precision and consistency. Despite the claims of greater accuracy measurements using WLI, the overall balance achieved by the SAVE results in fewer erroneous misses in practice, signifying its superiority within the real-world context. The SAVE has already proved its high efficiency with multiple models and subtypes, unlike WLI, meaning it can be deployed for the detection of advanced tumors, particularly where invasive deep-seated melanoma cases are more prevalent.

## Figures and Tables

**Table 1:** Results comparison between different models.

Image Modality	Class	Precision (in %)	Recall (in %)	F1 Score (in %)	mAP (in %)
YOLOv10					
WLI	ALM	85	82	83	81
	M in Situ	70	67	68	
	NM	87	69	76	
	SSM	79	63	70	
SAVE	ALM	83	83	83	92
	M in Situ	89	84	86	
	NM	95	86	90	
	SSM	84	85	84	
Faster R-CNN					
WLI	ALM	97	95	96	93
	M in Situ	98	93	96	
	NM	100	79	88	
	SSM	94	95	94	
SAVE	ALM	100	92	96	86
	M in Situ	90	89	90	
	NM	95	86	90	
	SSM	94	77	85	
Scaled YOLOv4					
WLI	ALM	82	74	78	68
	M in Situ	70	79	74	
	NM	88	66	76	
	SSM	86	81	83	
SAVE	ALM	80	84	82	78
	M in Situ	88	86	87	
	NM	82	80	81	
	SSM	87	82	84	
YOLOv7					
WLI	ALM	74	69	71	63
	M in Situ	67	64	65	
	NM	70	52	59	
	SSM	62	64	62	
SAVE	ALM	73	77	75	77
	M in Situ	82	78	80	
	NM	81	73	76	
	SSM	77	77	77	

## Data Availability

The data presented in this study are available in this article upon considerable request to the corresponding author (H.-C.W.).

## Supplementary Materials

The following supporting information can be downloaded at: https://www.mdpi.com/article/10.3390/diagnostics15060714/s1, Figure S1: Confusion matrix of YOLOv10: WLI; Figure S2: Loss graphs for YOLOv10: WLI; Figure S3: Confusion matrix of YOLOv4: SAVE; Figure S4: Loss graphs for YOLOv10: SAVE; Figure S5: Results of YOLOv10: WLI; Figure S6: Results of YOLOv10: SAVE; Figure S7: Confusion matrix for Faster-RCNN: WLI; Figure S8: Confusion matrix for Faster R-CNN: SAVE; Figure S9: Loss graph for scaled YOLOv4: WLI; Figure S10: Results of scaled YOLOv4:WLI; Figure S11: Loss graph for scaled YOLOv4: SAVE; Figure S12: The color difference before and after camera calibration; Figure S13: RMSEs between analog and measured spectra of each color block; Figure S14: LAB values of the simulated and observed colors; Table S1: RMSEs of the XYZ values before and after calibration. References [47,48,49,50,51,52,53,54,55,56,57] are cited in the supplementary materials.
