# Supplementary material for: Assessing the Efficacy of the Spectrum-Aided Vision Enhancer (SAVE) to Detect Acral Lentiginous Melanoma, Melanoma In Situ, Nodular Melanoma, and Superficial Spreading Melanoma: Part II
Source: Diagnostics (Basel). 2025 Mar 13;15(6):714. doi: 10.3390/diagnostics15060714 (PMC11941011; doi:10.3390/diagnostics15060714)
Supplement: Supplementary file 1 [file diagnostics-15-00714-s001.zip › diagnostics-3449903-supplementary.pdf]

diagnostics

MDPI

Article

Assessing the Efficacy of the Spectrum-Aided Vision Enhancer (SAVE) to Detect Acral Lentiginous Melanoma, Melanoma In Situ, Nodular Melanoma, and Superficial Spreading Melanoma: Part II—Supplementary Material

Teng-Li Lin [1], Riya Karmakar [2], Arvind Mukundan [2], Sakshi Chaudhari [3], Yu-Ping Hsiao [4,5], Shang-Chin Hsieh [6,*] and Hsiang-Chen Wang [2,7,*]

1 Department of Dermatology, Dalin Tzu Chi General Hospital, No. 2 Min-Sheng Rd., Dalin Town, Chiayi 62247, Taiwan; tanglilin1121@hotmail.com
2 Department of Mechanical Engineering, National Chung Cheng University, 168 University Rd., Min Hsiung, Chiayi 62102, Taiwan; karmakarriya345@gmail.com (R.K.); d09420003@ccu.edu.tw (A.M.)
3 Department of Computer Science, Sanjivani College of Engineering, Station Rd, Singapur, Kopargaon 423603, Maharashtra, India; sakshichaudharicomp@sanjivanicoe.org.in
4 Department of Dermatology, Chung Shan Medical University Hospital, No. 110, Sec. 1, Jianguo N. Rd., South Dist., Taichung City 40201, Taiwan; missyuping@gmail.com
5 Institute of Medicine, School of Medicine, Chung Shan Medical University, No. 110, Sec. 1, Jianguo N. Rd., South Dist., Taichung City 40201, Taiwan
6 Division of General Surgery, Department of Surgery, Kaohsiung Armed Forces General Hospital, 2 Zhongzheng 1st. Rd., Lingya District, Kaohsiung City 80284, Taiwan
7 Hitspectra Intelligent Technology Co., Ltd., Kaohsiung 80661, Taiwan
* Correspondence: sschin522@gmail.com (S.-C.H.); hcwang@ccu.edu.tw (H.-C.W.)

Abstract: This article provides the supplementary file for the article "Assessing the Efficacy of the Spectrum-Aided Vision Enhancer (SAVE) to Detect Acral Lentiginous Melanoma, Melanoma In Situ, Nodular Melanoma, and Superficial Spreading Melanoma: Part II". This supplement material presents additional insights and technical details that support the main findings of this study in the use of Spectrum Aided Vision Enhancer imaging for melanoma subtype detection. The motivation for this research is founded on the fact that early and precise diagnosis of melanoma, which is still challenging, is paramount. Conventional WLI suffers from limited spectral depth that inhibits early and effective melanoma diagnosis. Since WLI often fails to capture the full range of spectral data for the identification of subtypes of melanoma, it often leads to wrong identifications, hence the need for other imaging techniques.

Keywords: Skin cancer, Acral Lentignious Melanoma, Melanoma in Situ, Nodular Melanoma, Superficial Spreading melanoma, Hyperspectral imaging, band selection, spectrum-aided visual enhancer.

S1. Performance Evaluation of YOLOv10 on Melanoma Subtypes Detection

WLI

During training, several metrics were followed closely with respect to the YOLOv10 model on

A confusion matrix is also developed to understand, in greater detail, the performance of this model in practice. It shows true labels related to the four melanoma subtypes versus predicted classification. Large values on the diagonal indicate that YOLOv10 classifies with a small degree of misclassifications. The results here come from the efficiency of

YOLOv10 at finding distinctive features within melanomas with high precision and recall values.

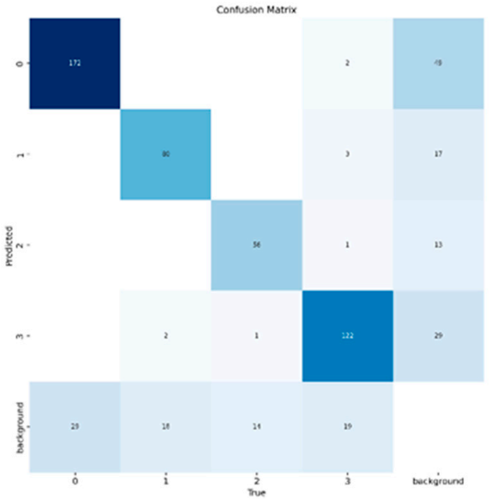

Figure S1: Confusion matrix of YOLOv10: WLI

Figure S1: The confusion matrix for the strong classification performance, with most of the true positives lying on the diagonal. Some misclassifications occurred, especially between class 1 and classes 0 and 3, since there are similarities that exist in the classes under WLI lighting conditions. Yet, the model fared well for both classes 0 and 3 with high sensitivity and a few errors.

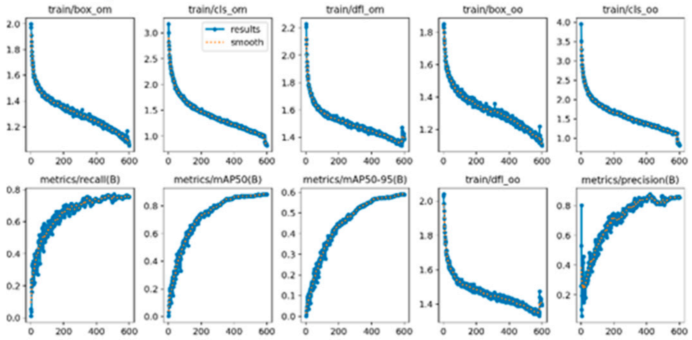

Figure S2: Loss graphs for YOLOv10: WLI

Figure S2: The training loss and metrics are almost smoothly improved in 600 epochs. The precision overscores of more than 0.8 while the recall is close to 0.8 reflect that the model has turned out to be increasingly confident and consistent. Improvement in mAP at 50% IoU and mAP50-95 reflects the robustness in localization and classification across object scales. Overall, YOLOv10 presents very good potential for automated melanoma detection with WLI; further optimization will be required to fix remaining errors in classification.

SAVE

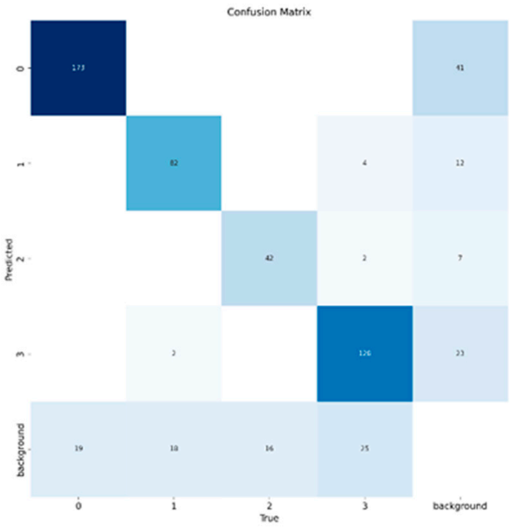

Figure S3:Confusion matrix of YOLOv4: SAVE

Figure S3: The confusion matrix exposes very good performance of the classification since most true positives lie in a diagonal manner. Misclassifications are few but do exist- especially between class 1 and classes 0 and 3, probably because of the similarities that exist under WLI lighting conditions. Even so, the model shows very high sensitivity with regard to classes 0 and 3 since there are fewer errors.

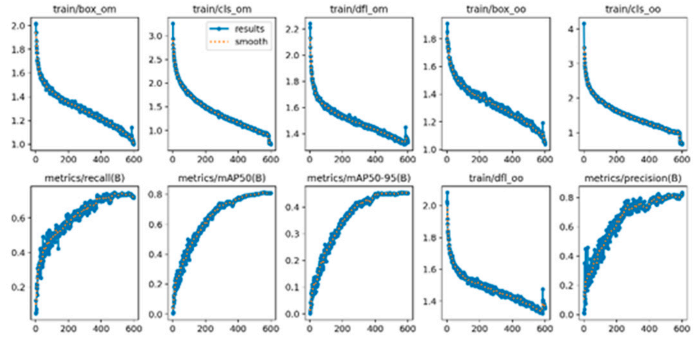

Figure S4: Loss Graphs for YOLOv10:SAVE

Figure S4: Training loss and performance metrics improved after 600 epochs. Precision is above 0.8 while recall approaches the same value, with all items demonstrating rising confidence and stability. Remarkable increases in mAP at 50% IoU and mAP50-95 ensure its robustness in detecting and classifying objects of varied scales.

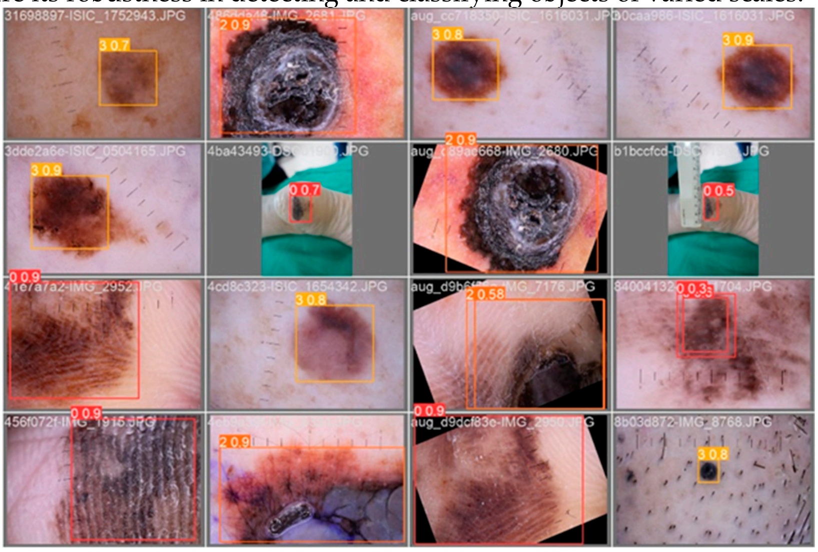

Figure S5: Results of YOLOv10:WLI

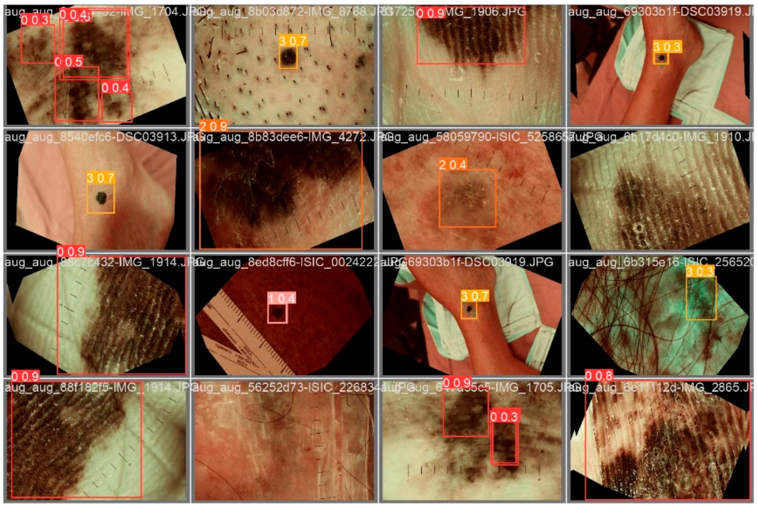

Figure S6: Results on YOLOv10: SAVE

On the whole, the YOLOv10 model demonstrated a very promising melanoma detection model based on WLI; however, some optimizations could be done related to the remaining classification errors.

S2. Performance Evaluation of Faster R-CNN on Melanoma Subtypes Detection

WLI

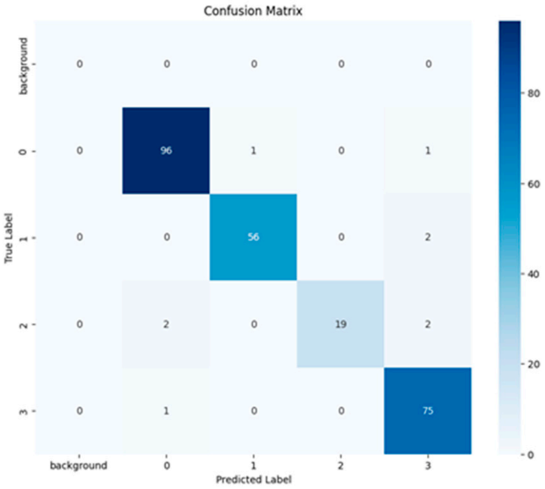

Figure S7: Confusion matrix for Faster-RCNN:WLI

Figure S7: Confusion matrix from the performance of the melanoma detection model using the object detection approach. It is evident that this performs better on the non-melanoma class, with 96 true positives and low false positives. The other classes, for example class 1, have a true positive count of 56, while class 2 has the lowest detection rates-it is sometimes confused with class 1-and class 3 was well detected at 75 correct predictions.

a. SAVE

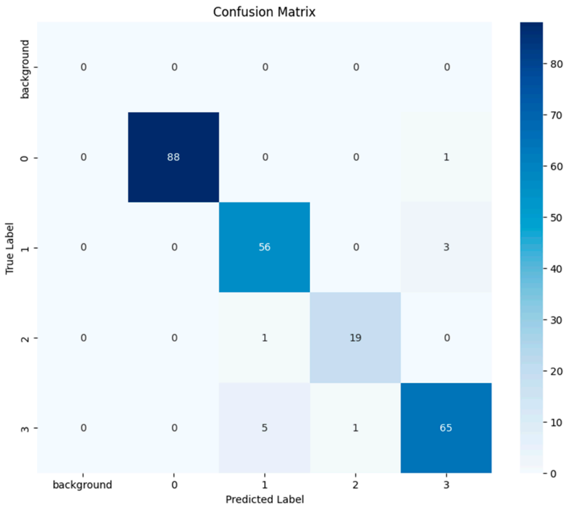

Figure S8: Confusion matrix for Faster R-CNN:SAVE

Figure S8: In the melanoma detection confusion matrix below, the model performs slightly worse in the detection of class 0 with 88 true positives compared to Figure S7. Class 1 also performs equally well with 56 correct detections, while increased confusion has taken place in class 3, where 5 misclassifications have occurred. Detection in class 2 remains similar, though with slight confusion between neighboring classes. In conclusion, while both models demonstrate strong performance in detecting melanoma and non-melanoma cases, WLI shows slightly higher accuracy overall, with fewer misclassifications across the different categories compared to SAVE.

S3. Performance Evaluation of Scaled YOLOv4 on Melanoma Subtypes Detection

WLI

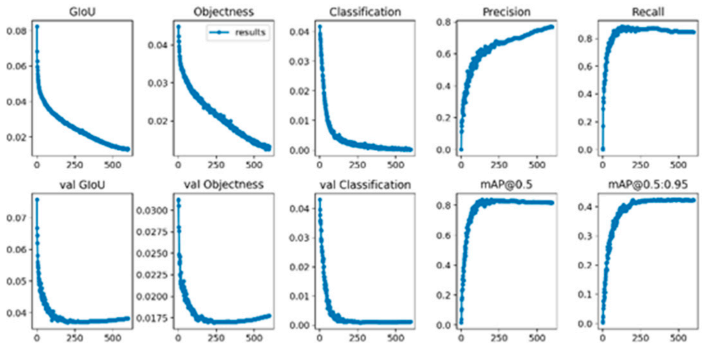

Figure S9: Loss Graph for Scaled YOLOv4: WLI

The graph reflects the performance and loss metrics for the melanoma detection model using WLI data. WLI relies on standard visible light, offering a clear view of the skin surface and visible irregularities. In these graphs, training and validation curves for GIoU, objectness, classification, precision, recall, and mean Average Precision (mAP) provide insights into the model's convergence, precision, and accuracy.

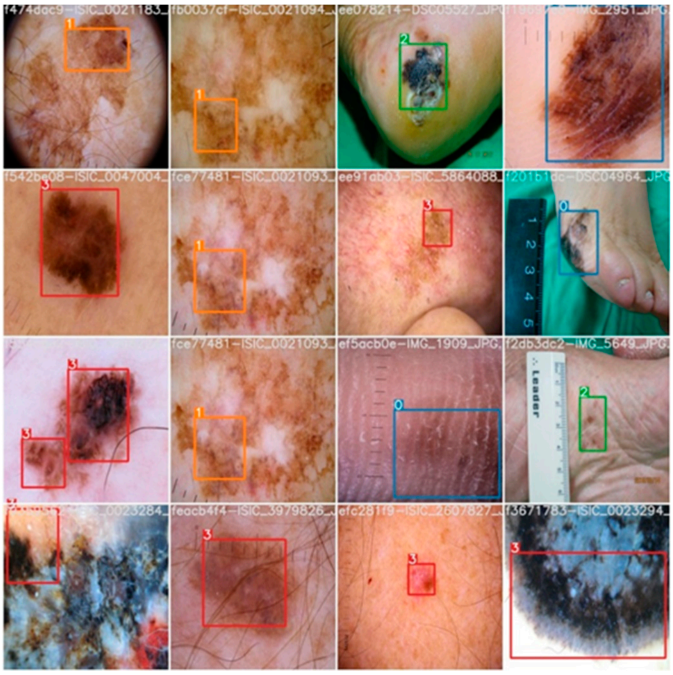

Figure S10: Results of Scaled YOLOv4:WLI

SAVE

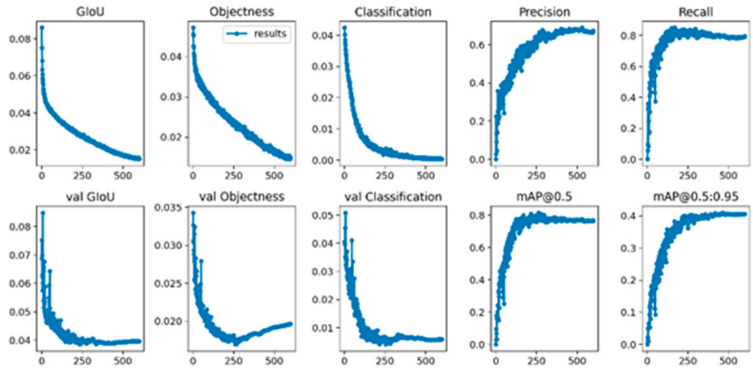

Figure S11: Loss Graph of Scaled YOLOv4: SAVE

This corresponds to performance metrics for melanoma detection using SAVE data, which likely enhances subsurface features like blood vessel structures and skin layers. The metrics here show similar trends, but the data may capture different aspects of melanoma lesions compared to WLI.

Both sets of graphs (WLI and SAVE) show typical curves for model training, with decreasing losses and improving precision and recall, indicating model optimization. The mAP curves give insight into the accuracy of the model at different IoU thresholds, with the model achieving better detection accuracy with SAVE, as seen in the smoother curve for mAP at 0.5 and 0.5:0.95.

S4. Evaluation Metrices

The evaluation metrics included in this particular study are Precision (P), Recall (R), mean Average Precision (mAP), F1-score and confusion matrix. Some of the concepts associated with these metrics are True Positive (TP), False Positive (FP), False Negative (FN). TP, just as the name suggests, are correct detection of ground truth bounding box. While FP are incorrect detections of a nonexciting class or misplaced detection of an object and FN are simply undetected ground-truth bounding box [1].

Precision is an evaluation metric that measures the number of instances that were correctly predicted [2]. It is given by equation (S1):

$$P = (\frac{TP}{TP+FP}) \times 100 \quad \text{(S1)}$$

High precision value indicates that the model has low rate of false positives. Having high precision reduces the chances of misclassifying any skin

Recall is known as positive rate and is a probability of actual positive instances that model identifies correctly [3]. It is given by equation (S2):

$$R = (\frac{TP}{TP+FN}) \times 100 \quad \text{(S2)}$$

F1-score is measure of predictive performance. It is calculated based on precision and recall of the model and is given by [4]:

$$F1 = \frac{2 \times Precision \times Recall}{Precision + Recall} \quad \text{(S3)}$$

The area under the precision-recall curve for a specific class in precision vs recall graph plotted for different threshold values is the average precision (AP). mAP is the mean of APs and is given by equation (S4):

$$AP = \int_0^1 Precision(Recall)\ dRecall \quad \text{(S4)}$$

S5. SAVE Results

The data generated through simulation were utilized for the initial evaluation of the system's performance. The characterization involved measuring their spectrum emissions, while also taking into account the sensitivities provided by the camera manufacturers. The spectral curves of the 24-color Macbeth Color Checker chart were utilized for both the training and validation sets of the samples. Table S1 displays the RMSE measurements for each of the 24 hues.

Table S1. RMSEs of the XYZ values before and after calibration.

S.no	Before calibration			After Calibration			RMSE	SD
	X	Y	Z	X	Y	Z		
1	10.96	9.92	4.63	11.14	9.87	4.26	0.24	0.30
2	38.74	35.80	18.65	38.57	35.94	18.66	0.13	0.08
3	16.62	19.07	24.13	16.48	18.79	24.11	0.18	0.17
4	10.33	12.86	4.62	10.16	13.03	4.85	0.19	0.19
5	24.05	23.87	31.55	24.16	24.07	31.60	0.13	0.08
6	30.12	42.15	32.40	30.10	42.17	32.42	0.02	0.002
7	38.10	30.24	4.28	38.04	30.37	4.22	0.09	0.04
8	11.70	11.47	25.90	11.64	11.37	25.91	0.07	0.02
9	29.01	19.91	9.62	29.20	19.78	9.60	0.13	0.08
10	8.26	6.49	9.63	8.06	6.49	9.86	0.18	0.17
11	34.15	44.06	8.44	34.15	44.02	8.53	0.06	0.01
12	47.99	44.55	6.05	48.05	44.34	6.17	0.15	0.11
13	6.82	5.79	21.07	6.90	5.91	21.00	0.09	0.04
14	14.55	23.55	7.22	14.58	23.51	7.12	0.07	0.02
15	21.08	12.25	3.57	21.01	12.28	3.65	0.06	0.01
16	58.40	60.69	7.54	58.38	60.79	7.42	0.09	0.04
17	28.98	19.54	20.67	28.94	19.52	20.66	0.02	0.002
18	12.81	19.01	28.54	12.84	19.10	28.56	0.05	0.01
19	82.12	88.54	67.20	82.31	88.73	67.51	0.24	0.30
20	54.74	58.92	45.52	54.28	58.40	44.75	0.60	1.89
21	33.08	35.73	27.24	33.26	35.82	27.54	0.21	0.23
22	18.18	19.62	14.94	18.86	20.31	15.62	0.68	2.43
23	9.13	10.01	8.13	8.56	9.26	7.21	0.76	3.04
24	2.87	3.19	2.39	3.10	3.35	2.68	0.23	0.27

Average	0.19	0.39

The calibration of the camera is a crucial component of the SAVE algorithm. Figure S12 displays the color disparity outcomes prior to and following calibration. Following the calibration of the camera, the color exhibited a striking resemblance to the color acquired by the spectrum analyzer, rendering the distinction challenging to perceive. Prior to camera calibration, the mean chromatic aberration of all 24 color blocks was 10.76. After calibrating the camera, the average chromatic aberration decreased to a minimum of 0.63.

S.no	Before Camera Calibration	Spectrometer	Chromatic Aberration	After Camera Calibration	Spectrometer	Chromatic Aberration
1			7.08			1.24
2			7.63			0.78
3			16.43			0.86
4			12.45			1.68
5			14.92			0.45
6			10.80			0.05
7			7.47			0.52
8			18.46			0.22
9			13.19			0.62
10			8.09			1.30
11			8.03			0.09
12			6.43			0.58
13			10.32			0.30
14			12.19			0.23
15			13.31			0.17
16			7.00			0.18
17			17.80			0.03
18			22.22			0.19
19			0.00			0.08
20			5.30			0.30
21			9.77			0.42
22			12.71			0.81
23			13.34			2.01
24			3.37			1.96
	Average Color Difference		10.76	Average Color Difference		0.63

Figure S12. The color difference before and after camera calibration

In Figure S12, the reflectance values of the six primary colors inside the 24-color block are illustrated. These colors are blue (13), red (15), green (14), yellow (16), magenta (17), and cyan (18). Based on the analysis of the 24-color blocks, it was noted that the red block exhibited the most significant disparity between the simulated and actual reflectance values, particularly across the longer wavelength range of 600 to 780 nm. One of the limitations of the study is considered to be this element. All of the remaining 23 color blocks exhibited RMSEs below 0.1, with the color black demonstrating the lowest RMSE of 0.015. The RMSE was merely 0.056, suggesting that the majority of the color could be replicated with precision.

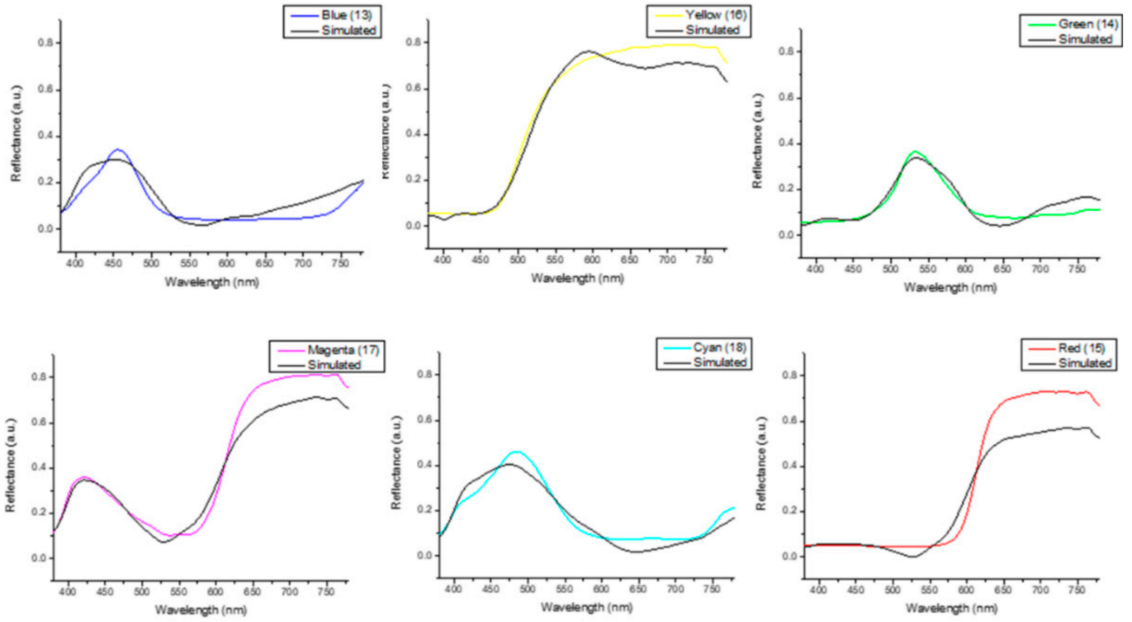

Figure S13. RMSEs between analog and measured spectra of each color block

RMSE values can be graphically and mathematically represented by calculating the disparity between simulated and measured colors. The representation of a color can be denoted as LAB, where L represents lightness, channel A, and channel B, respectively. The numerical definition of any color can be achieved by manipulating the values of L, A, and B. The L, A, and B values of the simulated and computed colors are depicted in Figure S14. The average color disparity was about 0.75, suggesting that the replicated color was visually precise.

Measured Color				Simulated Color				Color Difference
L	a	b	Color	L	a	b	Color	
37.61	13.65	24.56		37.64	11.87	22.60		1.32
66.48	14.68	31.10		66.37	15.47	30.58		0.73
50.44	-7.58	-6.44		50.72	-8.16	-6.09		0.75
42.80	-16.14	30.50		42.48	-14.04	31.19		1.50
56.16	5.70	-8.01		55.96	5.89	-8.51		0.43
70.99	-34.14	16.44		71.01	-34.00	15.65		0.41
61.97	32.36	66.76		61.85	33.07	65.32		0.80
40.20	6.07	-27.03		40.27	6.15	-26.67		0.27
51.59	46.04	27.52		51.72	44.60	27.21		0.52
30.62	18.70	-9.45		30.52	20.85	-8.70		1.46
72.24	-24.91	66.55		72.23	-25.21	66.79		0.13
72.46	17.04	75.67		72.59	16.33	76.17		0.49
29.18	13.90	-37.66		28.70	15.23	-38.14		0.81
55.59	-40.93	42.88		55.56	-41.82	42.54		0.42
41.66	53.78	34.95		41.60	54.34	34.25		0.51
82.26	1.48	87.73		82.21	1.96	87.67		0.27
51.29	46.36	1.08		51.30	46.12	0.95		0.10
50.80	-31.41	-12.85		50.66	-30.83	-13.20		0.42
95.47	-3.88	21.64		95.38	-3.70	22.54		0.52
80.96	-3.08	18.47		81.32	-3.39	17.74		0.63
66.38	-2.74	15.56		66.33	-3.33	15.44		0.69
52.18	-2.26	12.86		51.36	-2.59	12.85		0.91
36.47	-2.05	9.55		37.78	-3.18	8.64		2.00
21.40	-1.45	6.28		20.70	-2.87	7.50		2.02
							Average Color Difference	0. 75

Figure S14. LAB values of the simulated and observed colors.

4. State of the Art

Study	Used Dataset	Methodology	Pros	Cons	Originality	Results
**Diagnosis and prognosis of melanoma from dermoscopy images using machine learning and deep learning: a systematic review** (2024) SpringerLink	Various public dermoscopy image datasets	Systematic review of ML and DL applications in melanoma diagnosis	Comprehensive analysis of recent advancements	Limited to studies published up to 2024	Provides an updated synthesis of ML and DL techniques in melanoma diagnosis	Highlights the potential of DL models in improving diagnostic accuracy
**Melanoma diagnosis using deep learning techniques on dermatoscopic images** (2019) SpringerLink	Dermatoscopic images from ISIC archive	Development of a DL system for automatic melanoma detection	High reliability in detecting melanoma	Focused on a specific dataset; may require validation on diverse populations	Introduces a novel DL approach for melanoma detection	Achieved high sensitivity and specificity in melanoma recognition
**Melanoma skin cancer detection based on deep learning methods** (2024) SpringerLink	Dermoscopic images from HAM10000 dataset	Robust CNN-based method for classifying melanoma images	High classification accuracy	Requires large labeled datasets for training	Proposes a robust CNN architecture tailored for melanoma detection	Achieved superior accuracy compared to existing models
**Developing an efficient method for melanoma detection using CNN and k-means clustering** (2023) SpringerLink	Dermoscopic images from PH2 and ISIC datasets	Combination of Convolutional Neural Networks and k-means clustering for lesion segmentation and classification	Improved accuracy in melanoma detection	Computational complexity due to combined methods	Novel integration of CNN with clustering techniques	Demonstrated enhanced performance over traditional methods

**Detection for melanoma skin cancer through ACCF, BPPF, and CLF with SVM classifier** (2022) SpringerLink	Dermoscopic images from ISIC 2018 challenge	Ensemble models with Auto Correlogram Methods, Binary Pyramid Pattern Filter, and Color Layout Filter using SVM classifier	Enhanced feature extraction leading to better detection rates	Complexity in model training and parameter tuning	Combines multiple feature extraction methods with SVM for improved detection	Reported high sensitivity and specificity in melanoma detection
**Acral melanoma detection using dermoscopic images and convolutional neural networks** (2021) SpringerLink	Dermoscopic images from Yonsei University Health System	Novel deep learning model developed to classify skin cancer	Effective in detecting acral melanoma, a subtype prevalent in certain populations	May require further validation across diverse datasets	Focuses on a less-studied melanoma subtype using DL techniques	Achieved promising results in acral melanoma classification
**Fractional differentiation based image enhancement for automatic skin lesion detection** (2024) SpringerLink	Dermoscopic images from ISIC archive	Use of fractional differentiation for improved edge detection in skin lesion images	Enhanced edge detection leading to better segmentation	May introduce noise if not properly tuned	Applies fractional differentiation in the context of skin lesion detection	Improved segmentation accuracy, aiding in better diagnosis

References

1. Padilla, R.; Netto, S.L.; Da Silva, E.A. A survey on performance metrics for object-detection algorithms. In Proceedings of the 2020 International Conference on Systems, Signals and Image Processing (IWSSIP), Niteroi, Brazil, 1–3 July 2020; pp. 237–242.
2. Powers, D.M. Evaluation: from precision, recall and F-measure to ROC, informedness, markedness and correlation. *arXiv* **2020**, arXiv:2010.16061.
3. Sokolova M, Lapalme G. A systematic analysis of performance measures for classification tasks. *Inf. Process. Manag.* **2009**, *45*, 427-437.
4. Chicco, D.; Jurman, G. The advantages of the Matthews correlation coefficient (MCC) over F1 score and accuracy in binary classification evaluation. *BMC Genom.* **2020**, *21*, 6.
5. Naseri, H.; Safaei, A.A. Diagnosis and prognosis of melanoma from dermoscopy images using machine learning and deep learning: a systematic literature review. *BMC Cancer* **2025**, *25*, 75. https://doi.org/10.1186/s12885-024-13423-y.

6. Jojoa Acosta, M.F.; Caballero Tovar, L.Y.; Garcia-Zapirain, M.B.; Percybrooks, W.S. Melanoma diagnosis using deep learning techniques on dermatoscopic images. *BMC Med. Imaging* **2021**, *21*, 6. https://doi.org/10.1186/s12880-020-00534-8.
7. Jaber, N.J.F.; Akbas, A. Melanoma skin cancer detection based on deep learning methods and binary Harris Hawk optimization. *Multimedia Tools Appl.* **2024**. https://doi.org/10.1007/s11042-024-19864-8.
8. Moturi, D.; Surapaneni, R.K.; Avanigadda, V.S.G. Developing an efficient method for melanoma detection using CNN techniques. *J. Egypt. Natl. Cancer Inst.* **2024**, *36*, 6. https://doi.org/10.1186/s43046-024-00210-w.
9. Kavitha, P.; Ayyappan, G.; Jayagopal, P.; Mathivanan, S.K.; Mallik, S.; Al-Rasheed, A.; Alqahtani, M.S.; Soufiene, B.O. Detection for melanoma skin cancer through ACCF, BPPF, and CLF techniques with machine learning approach. *BMC Bioinform.* **2023**, *24*, 458. https://doi.org/10.1186/s12859-023-05584-7.
10. Abbas, Q.; Ramzan, F.; Ghani, M.U. Acral melanoma detection using dermoscopic images and convolutional neural networks. *Vis. Comput. Ind. Biomed. Art* **2021**, *4*, 25. https://doi.org/10.1186/s42492-021-00091-z.
11. Anber, B.; Yurtkan, K. Fractional differentiation based image enhancement for automatic detection of malignant melanoma. *BMC Med Imaging* **2024**, *24*, 231. https://doi.org/10.1186/s12880-024-01400-7.

Disclaimer/Publisher's Note: The statements, opinions and data contained in all publications are solely those of the individual author(s) and contributor(s) and not of MDPI and/or the editor(s). MDPI and/or the editor(s) disclaim responsibility for any injury to people or property resulting from any ideas, methods, instructions or products referred to in the content.
